# Uncovering gut microbiota-mediated indirect effects of antibiotic use on *Clostridioides difficile* transmission

**DOI:** 10.1017/ash.2023.378

**Published:** 2023-09-29

**Authors:** Camden Gowler, Prabasaj Paul, Mihnea Mangalea, Daniel Nkemzi, Hannah Wolford, Sujan Reddy, Alison Halpin, Lawrence McDonald, Rachel Slayton

## Abstract

**Background:** *Clostridioides difficile* and multidrug-resistant organisms (MDROs) pose challenges due to treatment complexities and substantial morbidity and mortality. Susceptibility to colonization with these organisms and potential onward transmission if colonized (ie, infectivity) is influenced by the human microbiome and its dynamics. Disruptive effects of antibiotics on the microbiome imply potential indirect effects of antibiotics on *C. difficile* colonization. Mathematical models can help explore the relative impact of key pathways linking antibiotic use to *C. difficile* colonization, including the relationship between population-level antibiotic use and colonization prevalence. **Methods:** We built a compartmental model of long-term *C. difficile* colonization prevalence of nursing home residents (though malleable for any MDRO), allowing interactions between the microbiome and the colonization process. Based on proportional abundance of microbial taxa, we classified individuals into high and low α diversity groups, each further stratified into uncolonized or colonized with *C. difficile*. The rate of transition from the high to low microbiome diversity group was proportional to the population-level rate of antibiotic use. Transmission dynamics followed a susceptible–infectious–susceptible framework with the possibility for increased susceptibility and infectivity for the low-diversity microbiome group. First, as a comparator, we used a “null model” in which microbiome diversity did not influence host susceptibility or infectivity. Next, we sampled from realistic (literature informed) parameter ranges to analyze how the microbiome mediates the effect of antibiotics on colonization in this population. **Results:** Our analysis suggests that antibiotic use can catalyze colonization with *C. difficile* through interactions with the host microbiome, resulting in a sharp increase in colonization with a modest increase in antibiotic use (Fig 1). Increasing the population-level antibiotic use by 5% led to a median 24% increase in long-term colonization prevalence in the model (Fig 2). In contrast, increasing susceptibility or infectivity rates by 5% resulted in slightly higher increases in total colonization (27% and 29%, respectively). However, there was considerable uncertainty around these estimates, with interquartile ranges of up to 20% for some parameters (Fig 2). **Conclusions:** Higher population-level antibiotic use likely increases colonization by *C. difficile* through indirect effects of the microbiome. The increased colonization burden attributable to increasing antibiotic use may be substantial. With high uncertainty around some estimates, conducting observational studies to better understand key colonization and microbiome parameters (eg, the relative increase in susceptibility or infectivity with lower microbiome diversity) is critical for future efforts to estimate the impact of antibiotic use on colonization with *C. difficile* and MDROs.

**Figure:**
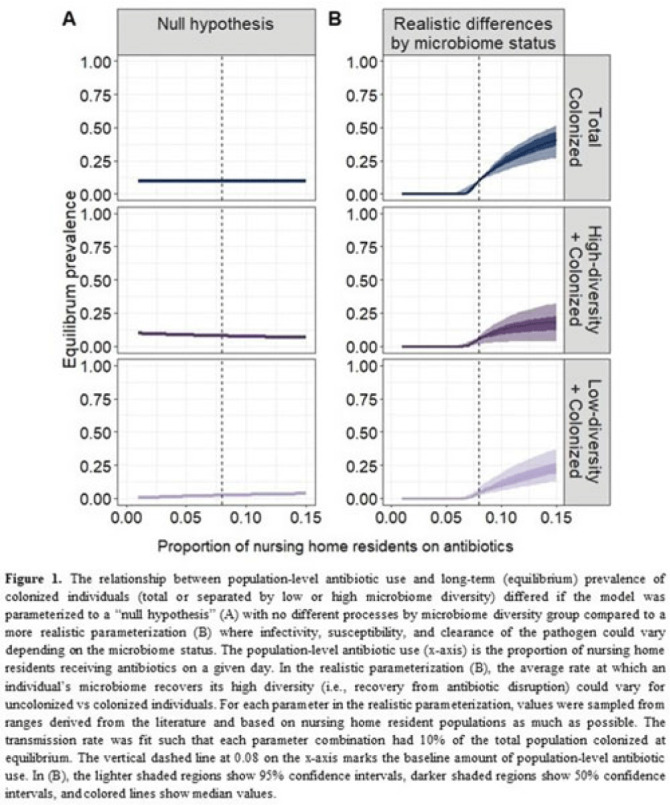

**Figure:**
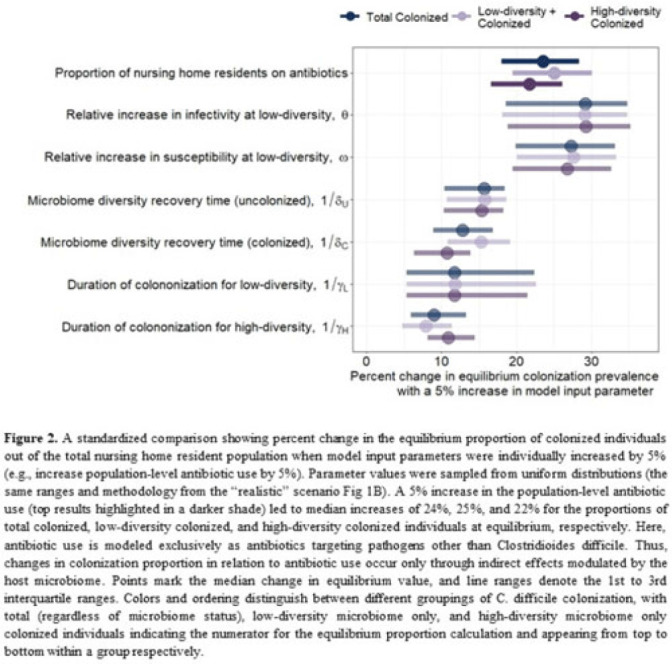

**Disclosures:** None

